# Neural heterogeneity as a unifying mechanism for efficient learning in spiking neural networks

**DOI:** 10.3389/fncom.2025.1661070

**Published:** 2025-11-07

**Authors:** Fudong Zhang, Jingjing Cui

**Affiliations:** 1 College of Intelligent Robotics and Advanced Manufacturing, Fudan University, Shanghai, China; 2 Department of Computer Science, City University of Hong Kong, Hong Kong, China

**Keywords:** neural heterogeneity, neural computation, spiking neural networks, deep learning, reservoir computing

## Abstract

The brain is a highly diverse and heterogeneous network, yet the functional role of this neural heterogeneity remains largely unclear. Despite growing interest in neural heterogeneity, a comprehensive understanding of how it influences computation across different neural levels and learning methods is still lacking. In this work, we systematically examine the neural computation of spiking neural networks (SNNs) in three key sources of neural heterogeneity: external, network, and intrinsic heterogeneity. We evaluate their impact using three distinct learning methods, which can carry out tasks ranging from simple curve fitting to complex network reconstruction and real-world applications. Our results show that while different types of neural heterogeneity contribute in distinct ways, they consistently improve learning accuracy and robustness. These findings suggest that neural heterogeneity across multiple levels improves learning capacity and robustness of neural computation, and should be considered a core design principle in the optimization of SNNs.

## 1 Introduction

Neuronal populations in the brain exhibit remarkable heterogeneity, even among neurons of the same physiological class. Emerging evidence suggests that this diversity is not merely noise but a fundamental feature of neural computation and processing (Markram et al., 1997; Marder and Goaillard, 2006; Brémaud et al., 2007; Gast et al., 2024). This heterogeneity spans structural, genetic, environmental, and electrophysiological dimensions, including spiking thresholds (Gast et al., 2024; Huang et al., 2016), membrane time constants (Perez-Nieves et al., 2021; Wu et al., 2025), external currents (Chen and Campbell, 2022; Montbrió and Pazó, 2020; Montbrió et al., 2015), electrical coupling strengths (Parker, 2003) and reset mechanisms (Leng and Aihara, 2020). These variations reflect the heterogeneity in cellular composition and network organization across brain regions (Billeh et al., 2020; Hawrylycz et al., 2012).

Although this neural heterogeneity might appear detrimental to the reliability of neural networks, both theoretical and empirical studies have demonstrated that it can, in fact, enhance information encoding, learning robustness, and task-specific computation (Chelaru and Dragoi, 2008; Padmanabhan and Urban, 2010; Mejias and Longtin, 2012). For example, diversity in membrane and synaptic time constants improves generalization in learning models (Perez-Nieves et al., 2021), while differences in spiking thresholds allow SNNs to flexibly gate, encode, and transform signals (Gast et al., 2024). However, focusing on a single source offers an incomplete picture of how heterogeneity shapes neural computation. Given its multifaceted nature, understanding the interplay between different types of heterogeneity remains an important and largely uncharted direction.

Building on prior theoretical frameworks (Ly, 2015), we classify neural heterogeneity into three main categories: external, network, and intrinsic. External heterogeneity stems from variations in input currents, which are experimenter-controlled and reflect properties of sensory stimuli rather than network structure (Chen and Campbell, 2022; Montbrió and Pazó, 2020; Montbrió et al., 2015; Goldobin et al., 2021; Luccioli et al., 2019). Network heterogeneity refers to structural diversity within the network, such as variability in synaptic connectivity or electrical coupling strengths (Marder and Goaillard, 2006; Oswald et al., 2009). Intrinsic heterogeneity, in contrast, originates from neuron-specific properties that are independent of network interactions (Padmanabhan and Urban, 2010). This classification provides a useful lens to systematically evaluate their computational contributions. While individual forms of heterogeneity have been explored in isolation, it remains unclear whether their computational effects are consistent across different learning methods and task domains. Addressing this gap could yield a more unified understanding of how heterogeneity shapes computation in both biological and artificial neural systems.

Previous studies have mainly explored neural heterogeneity in the special task and learning methods. For example, Ridge Least Square (RLS) learning method has demonstrated performance gains in basic curve-fitting tasks when neuron spiking thresholds or neural time constants are varied (Gast et al., 2024; Wu et al., 2025). To extend beyond such constrained scenarios, recent work has incorporated two broader learning methods (Nicola and Clopath, 2017). First, originating in the field of reservoir computing, First-Order, Reduced and Controlled Error (FORCE) learning method leverages the dynamics of high-dimensional recurrent systems to perform computations (Sussillo and Abbott, 2009; Schliebs et al., 2011; Buonomano and Merzenich, 1995). Learning is driven by an external supervisory signal that provides error feedback. This method expands the potential applications for more complicated target functions, such as chaotic system prediction, songbird generation and memory recall. Second, Surrogate Gradient Descent (SGD) (Neftci et al., 2019) enables SNNs to classify vision (Orchard et al., 2015; Lichtsteiner et al., 2008) and auditory (Cramer et al., 2020) stimuli, and this approach is well-suited for integration into modern SNNs architectures. While both methods have expanded the learning capacity of SNNs, it remains unclear whether neural heterogeneity offers consistent benefits across this range of learning methods from simple regression to high-dimensional classification.

In this study, we investigate the general computational role of neural heterogeneity across three representative forms: external current, synaptic coupling strength, and partial reset. These forms have been widely used in modeling studies, yet their impact remains poorly understood. We evaluate their effects across three complementary learning methods: RLS, FORCE, and SGD, and apply them to a diverse set of tasks ranging from curve fitting and network reconstruction to real-world speech and image classification. Our results demonstrate that neural heterogeneity, regardless of its origin, consistently improves both performance and robustness in SNNs. These findings underscore its fundamental computational advantages and offer a unified perspective on the functional role of heterogeneity in neural systems. We argue that heterogeneity is not merely a biological artifact but a key design principle for efficient and adaptable learning in next-generation SNNs. This work thus highlights a potentially unifying computational role of neural heterogeneity across multiple SNNs learning methods.

## 2 The basic SNN system

The Izhikevich (IK) model is a reduced form of the Hodgkin-Huxley-type neuron model, derived via bifurcation analysis (Izhikevich, 2007). Despite its reduced complexity, it preserves essential features of neuronal excitability through an adaptive quadratic mechanism. In this study, we incorporate external, network, and intrinsic heterogeneity by allowing three model parameters to vary across neurons.

External heterogeneity is introduced by assigning each neuron a unique external current, a method commonly employed in previous studies (Chen and Campbell, 2022; Montbrió and Pazó, 2020; Montbrió et al., 2015). This design mimics the inherent variability in sensory input processing in biological systems: sensory neurons receive non-uniform external drives due to differences in receptor sensitivity. For example, variations in the density of light-sensitive opsins in retinal cells or mechanosensitive channels in auditory hair cells lead to divergent responses to the same stimulus, which is well-documented in neuroscience (Chen and Campbell, 2022; Montbrió et al., 2015)

Network heterogeneity is captured by scaling the electrical coupling strengths, such that neurons receive varying degrees of network influence (Xue et al., 2014). This approach reflects extensive experimental evidence of heterogeneity in synaptic and electrical coupling (Marder and Goaillard, 2006; Oswald et al., 2009; Parker, 2003). By incorporating *g*_*i*_ heterogeneity our model replicates this structural diversity ensuring that network interactions align with the non-uniform connectivity patterns of real brains.

Intrinsic heterogeneity is implemented via the partial reset mechanism following spike generation, representing incomplete membrane repolarization (Leng and Aihara, 2020; Rospars and Lánskỳ, 1993). This mechanism allows neuron-specific post-spike dynamics, enhancing variability that arises intrinsically from the individual neuron's state. Similar approaches have been used to account for stochasticity and diversity in spiking behaviors (Bugmann et al., 1997; Kirst et al., 2009).

The network model for a population of coupled IK neurons is described by the following equations (Chen and Campbell, 2022)


$$\begin{array}{l} C\frac{\text{d}v_{i}}{\text{d}t}=k\left(v_{i}-v_{r}\right)\left(v_{i}-v_{t}\right)-w_{i}+I_{i}+g_{i}s\left(E-v_{i}\right), \end{array}$$ (1)

$$\begin{array}{l} \tau_{w}\frac{\text{d}w_{i}}{\text{d}t}=\beta \left(v_{i}-v_{r}\right)-w_{i}, \end{array}$$ (2)

$$\begin{array}{l} \text{if }v_{i}\geq v_{\text{peak }},\text{then }v_{i}\leftarrow v_{\text{reset }}+\theta_{i}\left(v_{i}-v_{\text{peak}}\right)\\ \text{ and }w_{i}\leftarrow w_{i}+w_{\text{jump}} \end{array}$$

where *v*_*i*_ denotes the membrane potential of the *i*th neuron and *w*_*i*_ its associated recovery variable, which governs spike-frequency adaptation. The input current is modeled as *I*_*i*_ = η_*i*_ + *I*_ext_(*t*), where η_*i*_ represents neuron-specific external input and *I*_ext_(*t*) is a global time-varying external drive. This formulation introduces external heterogeneity through the distributed input η_*i*_. Network heterogeneity is incorporated by assigning electrical coupling strengths *g*_*i*_, which scales the strength of recurrent input each neuron receives. The core biophysical parameters of each neuron include membrane capacitance *C*, the leakage parameter *k*, the resting potential *v*_*r*_, the spike threshold potential *v*_*t*_, the synaptic reversal potential *E*, the recovery variable time constant τ_*w*_, and the scaling factor β. When *v*_*i*_ reaches the spike cutoff *v*_peak_, it is reset according to a partial reset rule:


$$v_{\text{reset}}+\theta_{i}\left(v_{i}-v_{\text{peak}}\right),$$

where 0 < θ ≤ 1 is the reset coefficient for neuron *i*, introducing intrinsic heterogeneity. A value of θ_*i*_ = 0 corresponds to a full reset, while θ_*i*_ > 0 retains partial voltage history. Simultaneously, the adaptation variable undergoes a discrete jump by an amount *w*_jump_. The synaptic gating variable *s* lies between 0 and 1, representing the synaptic activation of each neuron. For analytical simplicity, we assume an all-to-all connectivity structure, which is a standard approximation widely used in prior studies (Montbrió et al., 2015; Byrne et al., 2020). The temporal dynamics of synaptic transmission are formally described by a first-order differential equation (Ermentrout and Terman, 2010)


$$\begin{array}{l} \tau_{s}\frac{\text{d}s}{\text{d}t}=-s+\frac{\tau_{s}s_{\text{jump}}}{N}\overset{N}{\underset{j=1}{\sum }}\underset{k\backslash t_{j}^{k}\leq t}{\sum }\delta \left(t-t_{j}^{k}\right), \end{array}$$ (3)

where δ(*t*) is the Dirac delta function, and $t_{j}^{k}$ represents the time of the *k*th spike of the *j*th neuron. τ_*s*_ is a decay time constant, and *s*_jump_ is the coupling strength. We consider the network system of Equations 1–3 as the basic components of the following SNNs.

## 3 Results

To introduce different sources of heterogeneity, we assume that the neuron-specific parameters {η_*i*_, *g*_*i*_, θ_*i*_} are distributed according to a Lorentzian probability distribution parameterized by a center and half-width-at-half-maximum, as described by the density function


$$\begin{array}{l} p\left(*\right)=\frac{1}{\pi }\frac{\Delta_{*}}{{\left[*-\bar{*}\right]}^{2}+\Delta_{*}^{2}}, \end{array}$$ (4)

where * = η, *g*, θ, denoting the three sources of heterogeneity respectively.

Here, Δ^*^ denotes the half-width-at-half-maximum of the Lorentzian distribution, which controls the spread of heterogeneity. Larger Δ^*^ values correspond to greater variability in the parameter. This means that Δ_η_ controls the range of neuron-specific external currents, Δ_*g*_ controls the spread of coupling strengths, and Δ_θ_ controls the variability of partial reset coefficients).

The Lorentzian distribution is chosen not only for mathematical tractability (facilitating mean-field analysis in Appendix A) but also for biological relevance: it captures the heavy-tailed variability of sensory input drives observed in empirical studies unlike Gaussian distributions that underestimate the range of real-world sensory neuron responses (Gast et al., 2024; Perez-Nieves et al., 2021).

### 3.1 Heterogeneity is needed in the RLSM learning network

We now examine the role of neural heterogeneity in enabling reliable input-output mapping within a reservoir computing framework. Reservoir computing is particularly well-suited for processing temporal data, as it employs a fixed, high-dimensional dynamical system to transform input signals into rich spatiotemporal representations. These internal dynamics can then be decoded using simple readout mechanisms. By constructing a reservoir composed of heterogeneous spiking neurons, we investigate how different forms of heterogeneity contribute to computational reliability and temporal pattern processing. This approach enables us to probe the network's capacity to encode, retain, and transform temporal information. Analysis of the network responses reveals that neuronal heterogeneity enhances the system's ability to process complex input patterns and supports more robust and reliable computation.

In this experiment, we evaluate the network's capacity for reliable input-output mapping when processing complex temporal patterns within a reservoir computing framework. Information transformation is a core computational function supported by the collective dynamics of recurrent neural populations (Vyas et al., 2020). Through coordinated activity, recurrently connected spiking neurons can extract salient features from input streams and generate temporally extended output signals as nonlinear transformations of those features. While most model parameters are calibrated to match the biophysical properties of hippocampal CA3 pyramidal neurons (Nicola and Campbell, 2013b), the appropriate range for the external current η_*i*_ remains uncertain. To address this, we employ a mean-field analysis to identify a suitable operating regime (see Appendix A for details).

**Figure 1:**
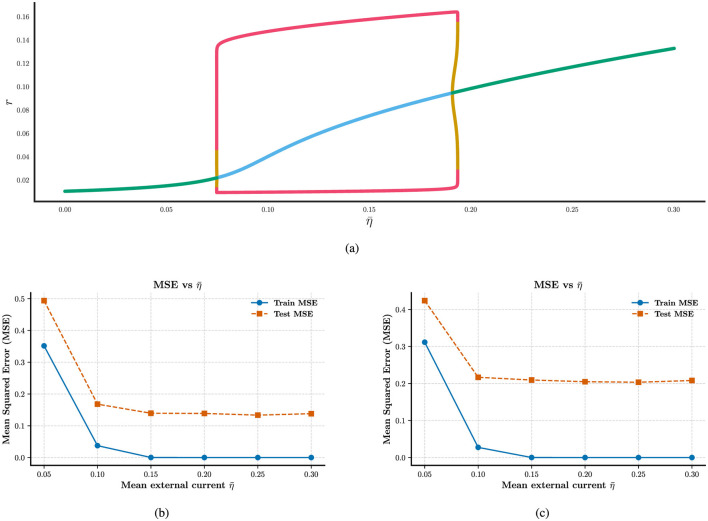
Identification of suitable external current for neural computation. **(a)** The bifurcation of the firing rate for the mean-field with mean external current $\bar{\eta }$. Green, blue, red, and mustard curves represent stable fixed points, unstable fixed points, stable limit cycles, and unstable limit cycles, respectively. **(b)** and **(c)** Training and testing MSE as a function of mean external current $\bar{\eta }$ for different target functions *y*_1_(*t*)and*y*_2_(*t*).

The mean-field model offers a reliable approximation of the bifurcation structure underlying neuronal population dynamics, a feature that is critical for understanding and optimizing reservoir computing performance. Using the software XPPAUT (Ermentrout and Mahajan, 2003), we numerically continued the mean-field **Equations 8–11** to obtain the bifurcation diagram shown in Figure 1a. This figure illustrates how the population firing rate *r* changes qualitatively with variations in the mean current $\bar{\eta }.$ As $\bar{\eta }$ increases, the system transitions through three distinct dynamical regimes: I. an asynchronous, quiescent regime, II. a synchronous, oscillatory regime, and III. an asynchronous, persistently active regime. To examine how these regimes affect network performance, we varies $\bar{\eta }$ in Figures 1b, c. And we used two target functions: a simple sinusoidal signal *y*_1_(*t*) = sin(12π*t*) and a more complex signal *y*_2_(*t*) = sin(12π*t*)sin24π*t* in Figures 1b, c. These functions were chosen to test the network's ability to model both periodic and non-periodic temporal dynamics. When the system is in the quiescent regime, the network fails to reproduce the target dynamics, primarily due to the low firing rates of individual neurons, which hinder effective information encoding and propagation. In contrast, once the system enters the oscillatory regime, both its responsiveness to external inputs and its ability to support dynamic information processing are markedly enhanced. Notably, performance remains comparably high in both the oscillatory and persistently active regimes. These observations suggest that coordinated neural activity plays a key role in computation. To promote effective learning, we therefore initialize the mean background current $\bar{\eta }$ just above the lower Hopf bifurcation point, ensuring the system operates in a dynamic and computationally favorable regime. Specifically, we initialize an all-to-all coupled network in the oscillatory regime, then apply a brief external stimulation to all neurons and record the network's response. Finally, we evaluate the network's ability to generate a time-dependent target output by training a single readout unit to decode the network's activity, while keeping the recurrent connectivity fixed (see Appendix A for details).

**Figure 2:**
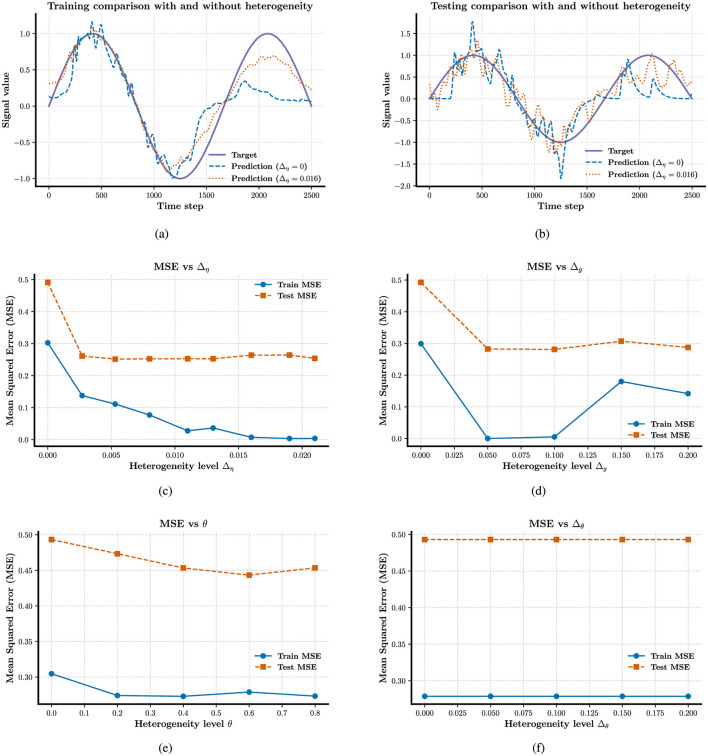
Enhancing the stability of neural computation using RLS in the presence of heterogeneity. **(a)** and **(b)** Shown are the curve fitting results on training and test datasets under conditions with and without external current heterogeneity. **(c–e)** Results about the MSE on both training and test sets varies as a function of external, network, and intrinsic heterogeneity. **(f)** MSE on training and test sets as a function of partial reset coefficient heterogeneity. All plots show mean MSE values across 50 trials, with standard deviations omitted for clarity.

Meanwhile, in Figures 2a, b, we observe that heterogeneity exerts a profound influence on the computational capability of the reservoir. When all forms of heterogeneity are removed, the network fails to perform accurate fitting, even under the initial parameter configurations. In Figures 2c–e, remarkably, reintroducing any single form of heterogeneity, among the three types considered, is sufficient to restore the network's computational functionality. We find that increasing the degree of heterogeneity does not lead to a significant reduction in computational error, in contrast, excessive heterogeneity harms to the neural computation in Figures 2d, e. We also find that the mean partial reset coefficient is more important than the width Δ_θ_ in neural computation, the latter not display a role in Figure 2f. While the degree of heterogeneity does not lead to a significant reduction in computational ability, the presence of heterogeneity induces a qualitative improvement in the network's ability to perform the task.

Our results further indicate that heterogeneity exerts a more pronounced effect on training accuracy than on test accuracy, as shown in the performance plots. This discrepancy may indicate a degree of overfitting. These findings suggest that conventional reservoir computing frameworks may be insufficient to fully capture the ways in which heterogeneity shapes the computational dynamics of spiking neural populations. This limitation motivates us to extend our investigation to more robust learning methods, such as the FORCE learning algorithm, which we explore in the following section.

### 3.2 Heterogeneity is needed in the FORCE learning network

The FORCE learning algorithm integrates real-time error feedback into the neuronal dynamics, making it well-suited for training SNNs to perform complex dynamical tasks. Our results show that, even without heterogeneity, adequate training performance can be achieved given carefully tuned hyperparameters. However, such success in homogeneous networks is highly sensitive to hyperparameter settings, suggesting a lack of robustness and adaptability. Prior research indicates that performance depends heavily on two key hyperparameters (Nicola and Clopath, 2017; Perez-Nieves et al., 2021). To examine whether heterogeneity can enhance learning robustness under mistuned conditions, we apply FORCE learning to SNNs tasked with reproducing diverse target trajectories. Details of our experimental setup and findings are provided in Appendix B.

**Figure 3:**
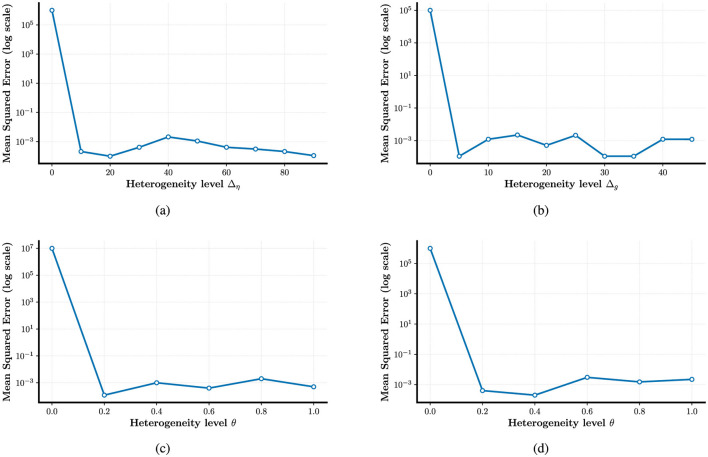
Enhancing the stability of neural computation using FORCE in the presence of heterogeneity. **(a)** Error response to external current (Δ_η_) heterogeneity for *G* = 2 × 10^3^, *Q* = 9 × 10^3^. **(b)** Error response to electrical coupling strengths (Δ_*g*_) heterogeneity for *G* = 2 × 10^3^, *Q* = 9 × 10^3^. **(c)** and **(d)** Effect of intrinsic reset (θ) heterogeneity on error for *G* = 2 × 10^3^, with *Q* = 4.5 × 10^3^ and *Q* = 1.35 × 10^4^, respectively.

**Figure 4:**
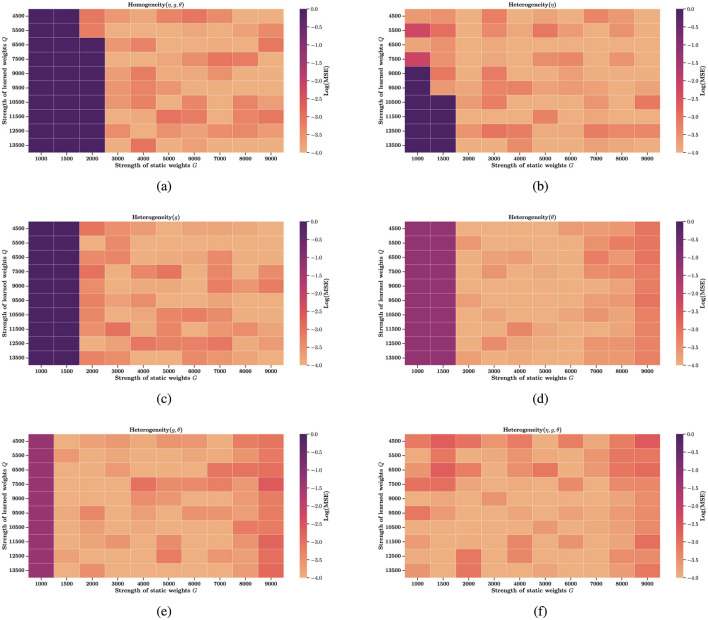
Heatmap of reconstruction error across learning hyperparameters. Each panel shows the log-scaled mean squared error (log(MSE)) as a function of learning hyperparameters *G* and *Q*. Dark purple regions (log(MSE) > 0) indicate failure to accurately reconstruct the target signal, while lighter regions represent higher computational precision. **(a)** Homogeneous network (no heterogeneity). **(b)** External input heterogeneity. **(c)** Network heterogeneity (e.g., synaptic variability). **(d)** Intrinsic neuronal heterogeneity. **(e)** Combined network and intrinsic heterogeneity. **(f)** Full heterogeneity: external, network, and intrinsic. All colorbars represent log(MSE).

In fact, some studies have explored the role of time constant heterogeneity in mitigating sensitivity to hyperparameters, but they focus on a single source of heterogeneity (Perez-Nieves et al., 2021). To generalize these findings, we extend the investigation to multiple forms of heterogeneity. In Figures 3a–d, we evaluate several hyperparameter configurations under which a homogeneous network fails to learn or reproduce the target trajectories. Remarkably, introducing any single source of heterogeneity, whether external, network, or intrinsic, restores the network's computational ability almost immediately. Figures 4a–d examine how performance, measured as log(MSE), varies with respect to different values of two key hyperparameters: *G*, the gain of the static recurrent weights (used to initialize the network in a chaotic regime), and *Q*, the coefficient of the learned weights in FORCE learning. Without heterogeneity, reducing *G* causes the system to fall out of the active regime, rendering it incapable of reproducing target dynamics for any *Q*. However, heterogeneity in external current (Δ_η_), electrical coupling Δ_*g*_, or partial reset coefficient (θ)significantly enlarges the viable region of the (*G, Q*) space where reliable learning is possible. In particular, Δ_η_ extends the lower boundary of *G* from 3,000 to 1,000 in certain configurations. Finally, Figures 4e, f explore the joint effects of multiple heterogeneities. Our results show that the combination of all three forms of heterogeneity exerts a synergistic effect, fully restoring computational performance across the entire tested range of *G* and *Q*. This highlights the powerful stabilizing role of heterogeneity in SNNs under FORCE learning.

We build upon prior findings focused on a single form of heterogeneity and present a more comprehensive characterization of its functional role. However, our current analysis is still largely limited to relatively simple SNNs architecture. To gain deeper insight into the impact of heterogeneity on neural computation within more powerful and complex models, we extend our investigation into the domain of deep learning. Specifically, we adopt SGD method to facilitate gradient-based training in deep SNNs. This approach enables us to systematically assess how various forms of heterogeneity influence computational performance under conditions that more closely resemble real-world tasks and deeper hierarchical network structures.

### 3.3 Heterogeneity is needed in the SGD learning network

We systematically investigated how neural heterogeneity influences task performance by training SNNs to approximate both simple and complex temporal dynamics. However, SNNs have also found applications in diverse domains such as image classification (Huang et al., 2023; Li et al., 2023) and speech recognition (Liu et al., 2023; Song et al., 2022). To further assess the impact of neural heterogeneity on task performance, we evaluated its influence on the ability of SNNs to classify both visual and auditory stimuli. We employ four benchmark datasets with varying degrees of temporal complexity: Neuromorphic MNIST (N-MNIST) and the DVS128 Gesture dataset for visual tasks, and the Spiking Heidelberg Digits (SHD) and Spiking Speech Commands (SSC) datasets for auditory tasks (see Appendix C for details). While many existing studies use the Leaky Integrate-and-Fire (LIF) model as the neuronal substrate (Wu and Maass, 2025; Shen et al., 2025), we adopt the Izhikevich (IK) model in our experiments due to its richer biological realism (Fitz et al., 2020; Dur-e Ahmad et al., 2012). Previous research has also shown that the IK model can achieve superior performance in certain contexts (Izhikevich, 2007; Izhikevich and Edelman, 2008). Additionally, using the same neural model as in Sections A and B ensures consistency throughout our study.

**Figure 5:**
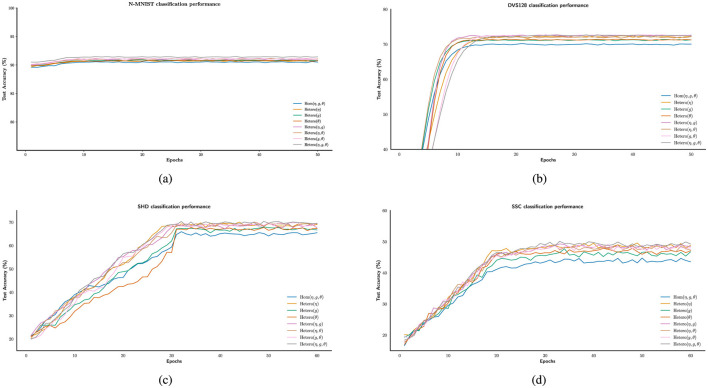
Enhancing the stability of neural computation using SGD in the presence of heterogeneity. **(a–d)** Example illustrating improved test accuracy on the N-MNIST, DVS128, SHD, and SSC dataset with and without different forms of heterogeneity-enabled SNN training.

In Figures 5a–d, the experimental results indicate that, although performance varies across datasets, heterogeneity consistently enhances the computational capacity of spiking neural populations. As the temporal complexity of the datasets increases, the accuracy improvement due to heterogeneity ranges from approximately 1% to 6%. This trend aligns with previous findings in LIF-based SNNs (Perez-Nieves et al., 2021), suggesting that heterogeneity may facilitate the encoding of temporal features. Table 1 summarizes the classification accuracies under different configurations of the heterogeneity parameters η, *g*, and θ, highlighting their respective contributions to performance improvements.

**Table 1:** Mean test accuracy (%, ± std) across datasets and configurations over 15 trials.

**Configuration**	**N-MNIST**	**DVS128**	**SHD**	**SSC**
Hom (η, *g*, θ)	90.0 ± 0.3	71.0 ± 0.7	65.0 ± 0.8	44.0 ± 0.5
Hetero (η)	90.7 ± 0.2	73.1 ± 0.7	68.9 ± 0.5	48.5 ± 0.8
Hetero (*g*)	90.5 ± 0.1	72.2 ± 0.4	67.0 ± 0.4	46.0 ± 0.5
Hetero (θ)	90.4 ± 0.2	72.5 ± 0.5	67.2 ± 1.0	47.0 ± 0.4
Hetero (η, *g*)	91.1 ± 0.4	73.5 ± 0.2	68.5 ± 0.7	48.5 ± 0.4
Hetero (η, θ)	91.2 ± 0.1	73.0 ± 0.5	69.1 ± 0.5	48.0 ± 0.2
Hetero (*g*, θ)	90.9 ± 0.3	**73.6** ± 0.7	69.0 ± 0.4	48.3 ± 0.3
Hetero (η, *g*, θ)	**91.3** ± 0.2	73.5 ± 0.8	**69.5** ± 0.8	**49.2** ± 0.2

Bold values denote the results with the highest average value in each dataset.

The test results across datasets reveal that heterogeneity plays a significant role in neural computation, contributing to improved computational accuracy. Notably, the combination of multiple forms of heterogeneity tends to exert a stronger facilitative effect. However, increasing the number of heterogeneous parameters does not necessarily lead to proportional performance gains. In several cases, similar accuracies were achieved with different numbers of heterogeneous components. For instance, on the SSC dataset, independent heterogeneity in parameter η yielded the same accuracy improvement as the joint heterogeneity of (η, *g*). This suggests that while heterogeneity is generally beneficial, the contribution of each source is not uniform. On the SHD dataset, we even observed that independent heterogeneity in η outperformed the combined heterogeneity of (η, *g*), indicating that multiple sources of heterogeneity do not always act synergistically. These findings highlight the need for careful consideration when selecting between single or combined forms of heterogeneity in neural computation models.

## 4 Discussion and conclusion

We investigate the role of heterogeneity in SNNs trained using methods ranging from traditional machine learning to state-of-the-art deep learning, considering three perspectives: external input, network structure, and intrinsic neuronal variability. Our findings consistently show that heterogeneity facilitates learning and enhances computational performance. Furthermore, the generalizability of its benefits across tasks and models supports its role as a fundamental mechanism in neural computation. These results offer promising directions for advancing biologically inspired neural system design.

A key mechanistic underpinning of heterogeneity's performance gains lies in its ability to enhance the dynamical richness of the network, as implied by the mean-field bifurcation analysis in Appendix A. The mean-field model reveals how the population firing rate transitions across distinct dynamical regimes, as the mean external current ($\bar{\eta }$) varies (Figure 1a). By introducing heterogeneity in external current (η_*i*_), coupling strength (*g*_*i*_), and partial reset (θ_*i*_), we expand the network's capacity to explore these regimes. For instance, in reservoir computing (Section 3.1), heterogeneity enables the network to move beyond the quiescent regime (where low firing rates hinder information encoding) and stably operate in oscillatory or persistently active regimes, stating that support robust temporal feature encoding. This dynamical expansion is critical for tasks requiring precise temporal processing, such as fitting complex sinusoidal signals (Section 3.1) and classifying time-varying stimuli in the DVS128, SHD, and SSC datasets (Section 3.3). Without heterogeneity, homogeneous networks remain confined to narrow dynamical ranges, limiting their ability to adapt to diverse input patterns.

Another theoretical basis for heterogeneity's benefits is its role in reducing sensitivity to hyperparameters, particularly evident in FORCE learning (Section 3.2). Homogeneous FORCE networks exhibit fragile performance, relying on tightly tuned hyperparameters G (gain of static recurrent weights) and Q (coefficient of learned weights) to maintain functional dynamics (Figure 4a). In contrast, introducing any single form of heterogeneity, whether in external current (Δ_η_), coupling strength (Δ_*g*_), or partial reset (θ), broadens the viable (*G, Q*) parameter space. For example, external current heterogeneity extends the lower boundary of *G* from 3,000 to 1,000 in certain configurations, while combining all three heterogeneities fully restores performance across the entire tested range of G and Q in Figures 4b–f. This hyperparameter robustness can be attributed to heterogeneity's ability to break symmetry in homogeneous networks: by introducing variability in neuron-specific properties, it reduces redundant neural activity and creates more distinct input-output mapping pathways. This symmetry breaking allows the network to efficiently utilize its high-dimensional dynamics, minimizing the risk of performance collapse due to slight hyperparameter deviations.

Our model's accuracy on real-world datasets such as DVS128 and SHD (e.g., a maximum accuracy of 73.6 ± 0.7% on DVS128, as shown in Table 1) has not yet reached that of state-of-the-art (SOTA) implementations. A major contributing factor is that our framework is built upon the Izhikevich neuron model, which, while providing a more biologically realistic description of spiking dynamics through the recovery variable (*w*_*i*_) that captures phenomena such as spike-frequency adaptation, has not been widely adopted in current deep SNN pipelines due to its higher computational cost and less optimized parameterization. As a result, our experiments focus primarily on establishing the fundamental computational role of neural heterogeneity, rather than achieving peak task-specific performance. Future work will incorporate our proposed principles into more advanced, SOTA architectures (e.g., hybrid convolutional SNNs or transformer-like event encoders) to further validate the generality of heterogeneity under high-performance settings.

Our findings align with and extend prior hypotheses about neural heterogeneity in biological systems. On one hand, we confirm the long-standing hypothesis that heterogeneity enhances learning robustness, which is a conclusion previously restricted to LIF networks and single heterogeneity types (Perez-Nieves et al., 2021; Mejias and Longtin, 2012). Our work broadens this insight by demonstrating that such robustness is universal across three distinct heterogeneity forms, three learning methods, and a variety of tasks from simple curve fitting to complex real-world classification. On the other hand, we also reveal that the relationship between the number and type of heterogeneity sources and performance is nonlinear: combined heterogeneity (e.g., joint variation in η, g, and θ) does not always outperform single-type heterogeneity. For instance, on the SHD dataset, independent heterogeneity in η alone yielded higher accuracy than the combined use of η and g. This finding suggests that biological systems may employ selective rather than accumulative heterogeneity, prioritizing specific forms according to task demands.

Nevertheless, our current results, obtained on moderately scaled networks, are sufficient to establish heterogeneity as a general and biologically plausible computational mechanism. We acknowledge that further Validation on more complex architectures and neuron models is necessary to fully assess the scalability and task-specific impact of heterogeneity. In future work, we aim to extend our framework toward SOTA network designs and larger datasets to bridge the gap between conceptual generality and high-performance realization.

In conclusion, our study provides systematic evidence that external, network, and intrinsic heterogeneity collectively enhance the learning capacity and robustness of SNNs. By linking these empirical findings to dynamical systems theory and hyperparameter sensitivity analysis, we strengthen the theoretical basis for heterogeneity as a core design principle, which bridges biological observations of neural diversity and engineering goals of optimizing SNN performance. This unifying perspective advances both our understanding of biological computation and our ability to construct more adaptive, efficient artificial neural systems.

## Data Availability

The original contributions presented in the study are included in the article/Supplementary material, further inquiries can be directed to the corresponding author.
