# Supplementary material for: Neural heterogeneity as a unifying mechanism for efficient learning in spiking neural networks
Source: Front Comput Neurosci. 2025 Nov 7;19:1661070. doi: 10.3389/fncom.2025.1661070 (PMC12634501; doi:10.3389/fncom.2025.1661070)
Supplement: Supplementary file 1 [file Presentation_1.pdf]

Supplementary material

APPENDIX A

A.1. Parameters, Model Equations and Mean-Field Model

The parameters used in our model are summarized in Table I and were selected to match the electrophysiological properties of hippocampal CA3 pyramidal neurons, as reported in [1]. To facilitate mathematical analysis and numerical simulation, we adopt a dimensionless formulation of the governing equations. For consistency and comparability, we follow the nondimensionalization approach presented in [1, 2].

$$\frac{\mathrm{d}v_i}{\mathrm{d}t} = v_i(v_i - \alpha) - w_i + \eta_i + I + g_i s(E - v_i), \tag{1}$$

$$\frac{\mathrm{d}w_i}{\mathrm{d}t} = a(bv_i - w_i), \tag{2}$$

$$\tau_s \frac{\mathrm{d}s}{\mathrm{d}t} = -s + \frac{\tau_s s_{\mathrm{jump}}}{N} \sum_{j=1}^{N} \sum_{k \backslash t_j^k \leq t} \delta(t - t_j^k), \tag{3}$$

$$\text{if } v_i \geq v_{\mathrm{peak}}, \text{then } v_i \leftarrow v_{\mathrm{reset}} + \theta_i(v_i - v_{\mathrm{peak}}) \\ \text{and } w_i \leftarrow w_i + w_{\mathrm{jump}}.$$

The corresponding variable transformations are provided in [2], where the authors derive mean-field equations under the assumption of heterogeneity in the parameter η. Building on this framework, we extend the analysis to incorporate all three sources of heterogeneity: external, network, and intrinsic. This leads to the following set of mean-field equations

$$\dot{r} = \frac{\Delta_\eta}{\pi} + 2rv - (\alpha + \bar{g}s)r, \tag{4}$$

$$\dot{v} = v(v - \alpha - \bar{g}s) - w + \bar{\eta} + I + \bar{g}sE - \pi^2 r^2, \tag{5}$$

$$\dot{w} = a(bv - w) + w_{\mathrm{jump}} r, \tag{6}$$

$$\tau_s \dot{s} = -s + \tau_s s_{\mathrm{jump}} r. \tag{7}$$

To simplify the analysis, we approximate the g_i by their respective means, $\bar{g}$. While it is theoretically possible to derive mean-field equations that explicitly incorporate heterogeneity in g_i, our findings suggest that considering heterogeneity in the external input η_i alone is sufficient to capture the essential population dynamics. This implies that the external current plays a dominant role in shaping the mean-field behavior of the system.

A.2. Numerical Experiments in RLSM Learning Network

We investigate how heterogeneity influences the network's response to external stimuli. Specifically, we apply brief input pulses to the network and assess its dynamic response. To obtain reliable statistics, this procedure is repeated over 60 trials, allowing us to evaluate the average effect of heterogeneity. Each simulation is run for over 1000ms, and the final network state is used as the initial condition for subsequent trials.

Table I. Parameters for the dimensional IK network based on [1, 3].

Parameter	Value	Parameter	Value
C	250 pF	k	2.5 nS/mV
v_r	-65 mV	v_t	-24.6 mV
g	-1 nS	E	0 mV
τ_w	200 ms	τ_s	4 ms
s_{jump}	0.8	w_{jump}	200 pA

We extract the neural firing rates X from the network responses, where $X \in \mathbb{R}^{N \times T}$ denotes the activity of N neurons over T time steps. These firing rates are then used to reconstruct and predict the target signal y via a linear readout: $\hat{y} = WX$. The readout weight matrix W is optimized to minimize the error between the predicted output $\hat{y}$ and the target signal y, using the RLS

$$\begin{aligned} W =& \mathrm{argmin} \sum_t \|\hat{y} - y\|_2^2 + \gamma \|w\|_2^2, \\ =& (XX^{\mathrm{T}} + \gamma I)^{-1} X y \end{aligned} \tag{8}$$

We provide examples for the target function on sin curves $y_1(t)$ and product of sin curves $y_2(t)$

$$y_1(t) = \sin(12\pi t), y_2(t) = \sin(12\pi t)\sin(24\pi t).$$

Figs. 1(a)–1(d) show the performance of SNNs trained using the RLS learning method on the target functions $y_1(t)$ and $y_2(t)$, evaluated on both the training and test sets.

APPENDIX B

B.1. Parameters and FORCE learning method

The FORCE learning algorithm is applied to a network composed of a single recurrent layer containing both LIF and IK neurons, following the approach in [4, 5]. Previous studies have examined the role of heterogeneity in LIF networks [6–9]. Here, we adopt a similar methodology to train the network to reproduce complex dynamical systems, including two-dimensional trajectories and the Lorenz system. In this framework, the synaptic weight matrix is defined as $W = GW^0 + Q\boldsymbol{\beta}\boldsymbol{\phi}^{\mathrm{T}}$. The static weights GW_0 are set to initialize the network into chaotic spiking. The learned component of the weights, $\boldsymbol{\phi}$, are determined online using a supervised learning method called Recursive Least Squares (ReLS). The vector $\boldsymbol{\beta}$ consists of fixed, randomly drawn components

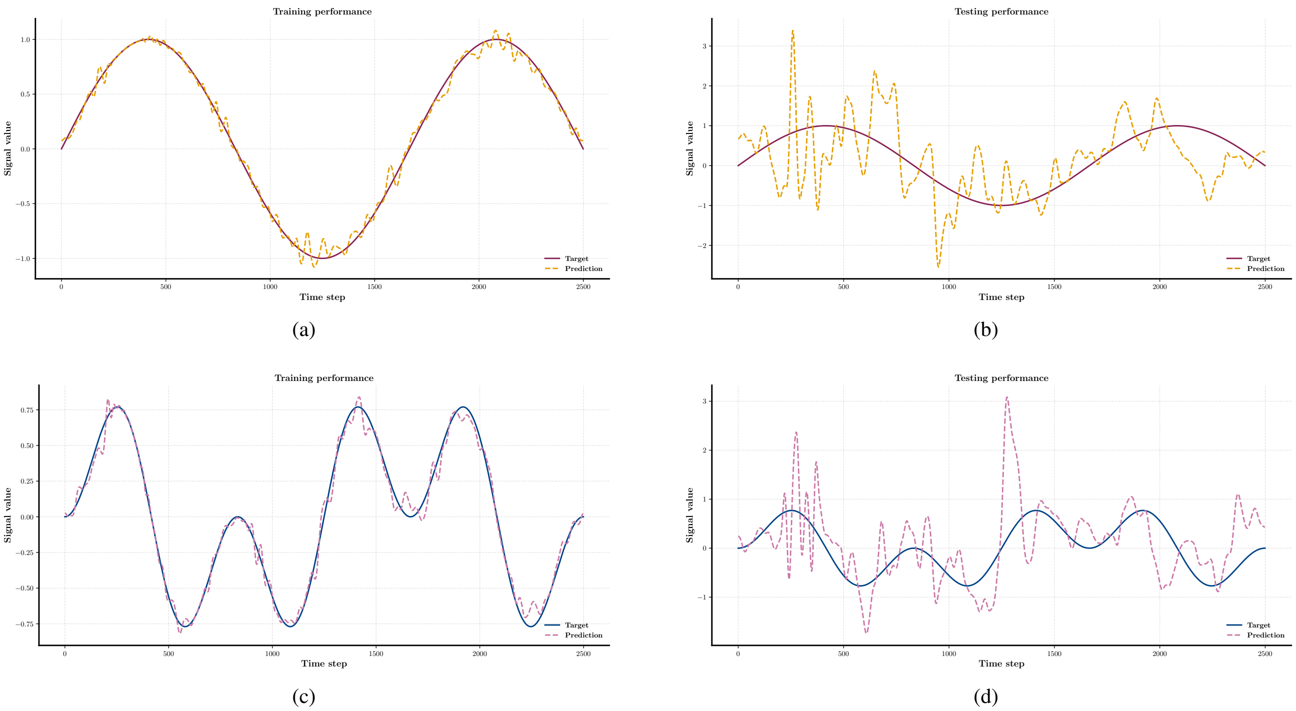

FIG. 1. **SNNs with RLS learning method**. **(a)-(d)** After reservoir computation with RLS, the performance of the network is evaluated separately for the two target functions $y_1(t)$ and $y_2(t)$ on both the training and test datasets. Each experiment is repeated five trails for demonstration purposes.

and determines how each neuron contributes to the readout. The product $\boldsymbol{\beta}\boldsymbol{\phi}^{\mathrm{T}}$ thus acts both as a feedback term and as a linear decoder of the network activity. The parameter $\boldsymbol{\beta}$ defines the tuning of individual neurons to the learned feedback signal. The hyperparameters G and Q balance the influence of the static and learned dynamics. The RLS learning rule minimizes the squared error between the network output and the target trajectory, enabling the network to learn and reproduce complex temporal dynamics in real time.

The equations differ slightly from Eqs. (1)–(3), particularly Eq. (1) and Eq. (3). The revised versions are as follows

$$C\frac{\mathrm{d}v_i}{\mathrm{d}t} = k(v_i - v_r)(v_i - v_t) - w_i + I_i, \tag{9}$$

$$\tau_w\frac{\mathrm{d}w_i}{\mathrm{d}t} = \beta(v_i - v_r) - w_i, \tag{10}$$

$$\text{if } v_i \geq v_{\mathrm{peak}}, \text{then } v_i \leftarrow v_{\mathrm{reset}} + \theta_i(v_i - v_{\mathrm{peak}}) \text{ and } w_i \leftarrow w_i + w_{\mathrm{jump}}$$

where $I_i = \eta_{\mathrm{bias}} + h_i$. η_{bias} is a background current, originally set at the spike threshold value in previous studies [4]. In our approach, we modify this by sampling η_{bias} from a Lorentzian probability distribution, allowing us to model the heterogeneity of external inputs. The spikes are filtered as in Eq. (3), but are subsequently mapped to specific synapse types

$$\tau_s\frac{\mathrm{d}s_i}{\mathrm{d}t} = -s_i + \frac{\tau_s s_{\mathrm{jump}}}{N}\sum_{j=1}^{N}\sum_{k\backslash t_j^k \leq t}\delta(t - t_j^k), \tag{11}$$

The synaptic currents, $h_i(t)$, are given by the equation

$$h_i = \sum_{j=1}^{N} W_{ij}s_j, \tag{12}$$

where the entries W_{ij} of the matrix W represent the strength of the postsynaptic current received by neuron i from neuron j, thereby controlling the magnitude of synaptic input each neuron receives. The primary objective of the network is to approximate the dynamics of an n-dimensional teaching signal, $\mathbf{x}(t)$, using the following equation

$$\hat{\mathbf{x}} = \boldsymbol{\phi}^{\mathrm{T}}\mathbf{s}. \tag{13}$$

The weights are dynamically adjusted over time by minimizing the squared error between the approximated and target dynamics, $\mathbf{e}(t) = \hat{\mathbf{x}}(t) - \mathbf{x}(t)$. The ReLS algorithm is used to update the decoders accordingly

$$\boldsymbol{\phi}(t) = \boldsymbol{\phi}(t-1) - \mathbf{e}(t)\mathbf{P}(t)\mathbf{s}(t), \tag{14}$$

$$\mathbf{P}(t) = \mathbf{P}(t-1) - \frac{\mathbf{P}(t-1)\mathbf{s}(t)\mathbf{s}(t)^{\mathrm{T}}\mathbf{P}(t-1)}{1 + \mathbf{s}(t)^{\mathrm{T}}\mathbf{P}(t-1)\mathbf{s}(t)}, \tag{15}$$

Table II. Parameters used for FORCE learning method in SNNs, following the implementation in [4].

Parameter	Value	Parameter	Value
C	250 pF	k	2.5 nS/mV
v_r	-60 mV	v_t	-20 mV
w_{jump}	200 pA	β	0 mV
τ_w	100 ms	τ_s	20 ms
η_{bias}	1000 pA	Δ_η	20 pA

During the training phase, the teaching signal $\mathbf{x}(t)$ is used to perform the ReLS update. During the testing phase, the teaching signal is removed, and the network evolves based on its intrinsic dynamics.

B.2. Numerical Experiments in FORCE Learning Network

Based on previous studies [4, 5], we provide the following parameter settings. The parameters used in our model are summarized in Table II. The initial synaptic weight matrix W_{ij}^0 is sparsely populated, with nonzero entries drawn from a normal distribution with zero mean and variance $(Np)^{-1}$, where $p = 0.1$ denotes the sparsity level. Neuronal membrane potentials are initialized from a uniform distribution $\mathcal{U}(v_r, v_t)$, and the vector $\boldsymbol{\beta}$ is sampled from a uniform distribution $\mathcal{U}(-1, 1)$. For the ReLS algorithm, the network is initialized with $\boldsymbol{\phi}(0) = 0, \mathrm{P}(0) = \mathbf{E}\lambda^{-1}$, where $\mathbf{E}$ is an N-dimensional identity matrix. Other parameters shown in Table. We use this learning method to mimic many complex target equations. We provide examples for the target function on sin curves $y_1(t)$, sawtooth curves $y_2(t)$, product of sin curves $y_3(t)$ and Van der Pol oscillator $y_4(t)$

$$\begin{aligned} y_1(t) &= \sin(10\pi t) \\ y_2(t) &= \mathrm{asin}(\sin(10\pi \mathrm{t})) \\ y_3(t) &= \sin(4\pi t)\sin(6\pi t)\sin(14\pi t), \end{aligned}$$

and we consider Van der Pol oscillator is given by

$$\ddot{y}_4(t) = \mu(1 - y_4^2)\dot{y}_4 - y_4,$$

where $\mu = 0.5$ is rescaled in space to lie within $[-1, 1]^2$ and speed up in time by a factor of 20. In Figs. 2(a)–2(d) show the performance of SNNs trained using the FORCE learning method on the target functions $y_1(t)$, $y_2(t)$, $y_3(t)$ and $y_4(t)$.

APPENDIX C

C.1. Model and SGD Learning Method

Previous research employed the leaky integrate-and-fire (LIF) neuron model in their simulations [5]. Following their approach, we adopt the IK neuron model instead.The parameters used in our model are summarized in Table III. In this model, the membrane potential of the ith in the lth layer, denoted as $v_i^{(l)}(t)$, evolves over time according to the model dynamics

Table III. Parameters used for SGD learning method in SNNs, following the implementation in [2].

Parameter	Value	Parameter	Value
α	0.6215	τ_s	2.6
a	0.0077	b	-0.0062
w_{jump}	0.0189	E	1
v_{peak}	200	v_{reset}	-200
η	1	Δ_η	0.1
g	1.2308	Δ_g	0.12308
θ	0.5	Δ_θ	0.05

$$\frac{\mathrm{d}v_i^{(l)}}{\mathrm{d}t} = v_i^{(l)}(v_i^{(l)} - \alpha) - w_i^{(l)} + I_i^{(l)},$$

where $I_i^{(l)} = \eta_i^{(l)} + g_i^{(l)} s_i^{(l)}(E^{(l)} - v_i^{(l)})$, spikes emitted by the jth neuron in layer $l-1$ at discrete times $\{t_j^{(l-1,\zeta)}\}$ are represented as a spike train $H_j^{l-1}(t)$, defined as

$$H_j^{(l-1)}(t) = \sum_\zeta \delta(t - t_j^{(l-1,\zeta)}).$$

The input current $s_i^{(l)}$ is computed from the spike trains of all presynaptic neurons j connected to neuron i, and is given by

$$\tau_s \frac{\mathrm{d}s_i^{(l)}}{\mathrm{d}t} = -s_i^{(l)} + \sum_j F_{ij}^{(l)} H_j^{(l-1)} + \sum_j R_{ij}^{(l)} H_j^{(l)},$$

where $F_{ij}^{(l)}$ denotes the feedforward synaptic weight from neuron j in layer $l-1$ to neuron i in layer l and $R_{ij}^{(l)}$ represents the recurrent synaptic weight between neurons j and i within layer l. The above equations can be compactly expressed in matrix form as follows

$$\frac{\mathrm{d}\mathbf{v}^{(l)}}{\mathrm{d}t} = \mathbf{v}^{(l)} \circ (\mathbf{v}^{(l)} - \alpha) - \mathbf{w}^{(l)} + \mathbf{I}^{(l)}, \tag{16}$$

$$\frac{\mathrm{d}\mathbf{w}^{(l)}}{\mathrm{d}t} = a \cdot \left(b \cdot \mathbf{v}^{(l)} - \mathbf{w}^{(l)}\right), \tag{17}$$

$$\tau_s \frac{\mathrm{d}\mathbf{s}^{(l)}}{\mathrm{d}t} = -\mathbf{s}^{(l)} + \mathbf{F}^{(l)}\mathbf{H}^{(l-1)} + \mathbf{R}^{(l)}\mathbf{H}^{(l)}, \tag{18}$$

$$\begin{aligned} \mathbf{v}^{(l)} &\leftarrow \mathbf{v}^{(l)} \cdot (\mathbf{1} - \boldsymbol{\delta}^{(l)}) \\ &\quad + \boldsymbol{\delta}^{(l)} \circ \left[\mathbf{v}_{\text{reset}} + \boldsymbol{\theta} \circ \left(\mathbf{v}^{(l)} - \mathbf{v}_{\text{peak}}\right)\right] \\ \mathbf{w}^{(l)} &\leftarrow \mathbf{w}^{(l)} + w_{\text{jump}} \cdot \boldsymbol{\delta}^{(l)} \end{aligned}$$

where $\mathbf{v}^l = [v_1^l, \ldots, v_N^l]^{\mathrm{T}}, \mathbf{w}^l = [w_1^l, \ldots, w_N^l]^{\mathrm{T}}, \mathbf{s}^l = [s_1^l, \ldots, s_N^l]^{\mathrm{T}}, \boldsymbol{\theta}^l = [\theta_1^l, \ldots, \theta_N^l]^{\mathrm{T}}$ and $\mathrm{I}^l = [I_1^l, \ldots, I_N^l]^{\mathrm{T}}$. $\mathbf{1}$

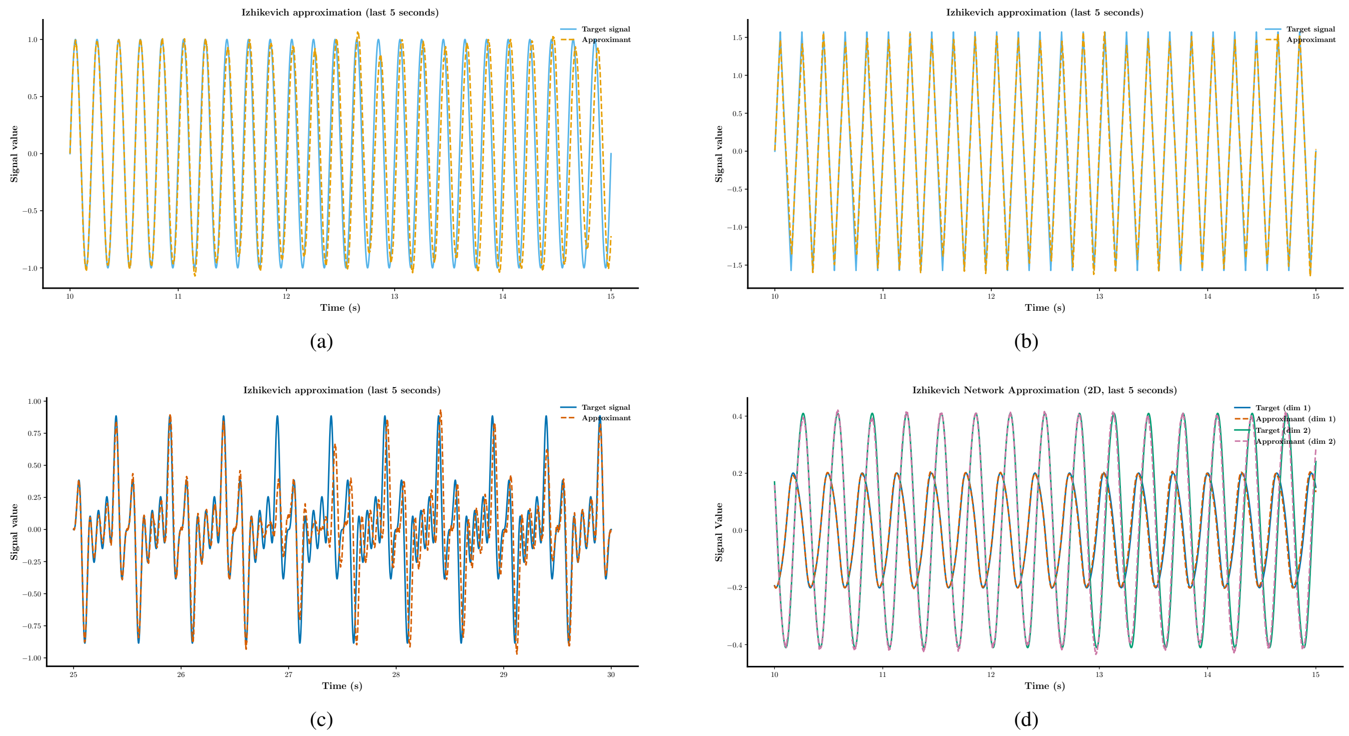

FIG. 2. **SNNs with FORCE learning method**. **(a)-(d)** After reservoir computation with RLS, the performance of the network is evaluated separately for the four target functions $y_1(t)$, $y_2(t)$, $y_3(t)$, and $y_4(t)$ on both the training and test datasets.

is an N-dimensional identity vector. $\boldsymbol{\delta}^{(l)} = \mathbb{I}\left[\mathbf{v}^{(l)} \geq v_{\text{peak}}\right]$, where $\mathbb{I}[\cdot]$ denotes the element-wise indicator function.

A spiking layer consists of three cascaded sub-components: input current integration, membrane potential dynamics, recovery variable updates, and spike generation. Following previous studies, we stack L such spiking layers to construct a deep SNN, analogous to conventional deep neural networks (DNNs), and train it using gradient-based optimization. However, the spike generation process is inherently non-differentiable, making standard backpropagation inapplicable. To address this, and consistent with prior work, we employ the BackPropagation Through Time (BPTT) algorithm to update the network parameters, namely,

$$\sigma(\mathbf{v}^{(l)}) = \frac{\mathbf{v}^{(l)}}{1 + \mathrm{k}|\mathbf{v}^{(l)}|}. \tag{19}$$

where surrogate steepness k is 100. Thus, gradient descent can be applied to optimize the synaptic weight matrices $\mathbf{F}^{(l)}$ and $\mathbf{R}^{(l)}$, analogous to standard deep learning. Each parameter is optimized individually for each neuron, consistent with our experimental setup. Training is performed using automatic differentiation in PyTorch, with the Adam optimizer (learning rate of 10^{-3} and $\beta_1 = 0.9, \beta_2 = 0.999$). To enhance classification performance, we adopt a max-over-time loss, which selects the maximal membrane potential across the entire time window in the readout layer. This value is then used to compute the cross-entropy loss for supervised learning

$$\mathcal{L}_{CE} = -\log \frac{\exp\{\arg\max_t o^{(l)}_{\text{target}}(t))\}}{\sum_i \exp\{\arg\max_t o^{(l)}_i(t)\}} \tag{20}$$

where target refers to the index of the readout neuron corresponding to the correct label for a given input sample. All neuronal states include membrane potential $\mathbf{v}^{(l)}(0), \mathbf{w}^{(l)}(0)$ and $\mathbf{s}^{(l)}(0)$ are initialised to $\mathbf{0}$. The initial feedforward and recurrent weight matrices, $\mathbf{F}^{(l)}$ and $\mathbf{R}^{(l)}$ are independently sampled from a uniform distribution $\mathcal{U}(-\mathrm{n}^{-\frac{1}{2}}, \mathrm{n}^{-\frac{1}{2}})$ where n denotes the number of incoming connections to each neuron, following standard initialization practices [10].

C.2. Introduction of the Datasets

We evaluate our model on five distinct datasets, each exhibiting varying levels of complexity. The N-MNIST and DVS128 Gesture datasets contain visual stimuli, while the SHD and SSC datasets are auditory in nature.

The N-MNIST and DVS128 Gesture datasets are derived from neuromorphic vision sensors that produce spike-based data. For N-MNIST, spiking activity is generated by moving a neuromorphic sensor across static images of handwritten digits (0–9), resulting in a dataset comprising 10 classes.

The images are low-resolution (28×28), making the dataset compact and easily accessible, while also enabling fast processing and rapid prototyping during algorithm development. In contrast, the DVS128 Gesture dataset contains event-based recordings of 11 distinct hand gestures performed by 29 subjects under three different lighting conditions. Each subject performs a sequence of 11 gestures, with each gesture lasting approximately 6 seconds.

In contrast, the auditory tasks SHD and SSC exhibit a much richer temporal structure. Both datasets are derived from corresponding audio corpora, with spike trains generated across 700 input channels using Lauscher, an artificial cochlea model. The SHD dataset comprises approximately 10,000 high-quality, temporally aligned studio recordings of spoken digits (0–9) in both German and English. Recordings are available from 12 distinct speakers, two of whom appear exclusively in the test set to assess generalization. The SSC dataset is based on Google's Speech Commands release and contains recordings of 35 word categories from a substantially larger and more diverse group of speakers. Unlike SHD, SSC was recorded under less controlled conditions, introducing greater variability and realism.

In all cases, we follow the train/test splits provided by the original authors of each dataset. The number of input neurons is adjusted for each dataset to optimize performance.

[1] W. Nicola and S. A. Campbell, Journal of computational neuroscience **35**, 87 (2013).

[2] L. Chen and S. A. Campbell, Journal of Computational Neuroscience **50**, 445 (2022).

[3] W. Nicola and S. A. Campbell, Frontiers in computational neuroscience **7**, 184 (2013).

[4] W. Nicola and C. Clopath, Nature communications **8**, 2208 (2017).

[5] N. Perez-Nieves, V. C. Leung, P. L. Dragotti, and D. F. Goodman, Nature communications **12**, 5791 (2021).

[6] C. Huang, A. Resnik, T. Celikel, and B. Englitz, PLoS computational biology **12**, e1004984 (2016).

[7] C. Ly, Journal of computational neuroscience **39**, 311 (2015).

[8] J. Mejias and A. Longtin, Physical Review Letters **108**, 228102 (2012).

[9] J. Lengler, F. Jug, and A. Steger, PloS one **8**, e80694 (2013).

[10] Y. LeCun, L. Bottou, G. B. Orr, and K.-R. Müller, in *Neural networks: Tricks of the trade* (Springer, 2002) pp. 9–50.
